# Bayesian Variable Selection in Cost-Effectiveness Analysis

**DOI:** 10.3390/ijerph7041577

**Published:** 2010-04-06

**Authors:** Miguel A. Negrín, Francisco J. Vázquez-Polo, María Martel, Elías Moreno, Francisco J. Girón

**Affiliations:** 1 Department of Quantitative Methods, University of Las Palmas de Gran Canaria, Faculty of Economics, Campus de Tafira, E-35017 Las Palmas de G.C. Canary Islands, Spain; E-Mails: mnegrin@dmc.ulpgc.es (M.A.N.); mmartel@dmc.ulpgc.es (M.M.); 2 Department of Statistics and Operation Research, University of Granada, Campus Fuentenueva, E-18071 Granada, Spain; E-Mail: emoreno@ugr.es; 3 Department of Statistics and Operation Research, University of Málaga, Campus de Teatinos, E-29071 Málaga, Spain; E-Mail: fj_giron@uma.es

**Keywords:** variable selection, Bayesian analysis, cost-effectiveness, BIC, Intrinsic Bayes Factor, Fractional Bayes Factor, subgroup analysis

## Abstract

Linear regression models are often used to represent the cost and effectiveness of medical treatment. The covariates used may include sociodemographic variables, such as age, gender or race; clinical variables, such as initial health status, years of treatment or the existence of concomitant illnesses; and a binary variable indicating the treatment received. However, most studies estimate only one model, which usually includes all the covariates. This procedure ignores the question of uncertainty in model selection. In this paper, we examine four alternative Bayesian variable selection methods that have been proposed. In this analysis, we estimate the inclusion probability of each covariate in the real model conditional on the data. Variable selection can be useful for estimating incremental effectiveness and incremental cost, through Bayesian model averaging, as well as for subgroup analysis.

## 1. Introduction

Econometric literature shows that modelling questions such as risk, resource use and the outcomes of alternative medical treatments is normally based on the use of covariates in regression models applied to microdata [1–6]. Several recent papers have proposed the use of covariates for the comparison of technologies through cost-effectiveness analysis (CEA). Hoch *et al*. [7] were pioneers in this research, showing that the use of regression analysis could produce more accurate estimates of treatment cost-effectiveness, by modelling the net monetary benefit in terms of covariates. Willan *et al*. [8] directly considered costs and effects jointly, assuming a bivariate normal distribution. Vázquez-Polo *et al*. [9] used an asymmetric framework in which costs are accounted for by effects, but effects, on the other hand, are not affected by cost. In a subsequent work, Vázquez-Polo *et al*. [10] proposed a general framework where effectiveness can be measured by means of a quantitative or a binary variable. In this study, costs were also analyzed taking into account the presence of a high degree of skewness in the distribution. Nixon and Thompson [11] developed Bayesian methods whereby costs and effects are considered jointly, and allowed for the typically skewed distribution of cost data by using Gamma distributions. Manca *et al*. [12] also included covariates in a multilevel framework for multicentre studies.

One of the aims of regression models in CEA is to infer causal relationships between a dependent variable (cost or effectiveness) and the variable of interest (e.g., medical treatment). Other variables, known as control variables, are included to minimize the bias and uncertainty of the estimation when there are differences in the baseline characteristics of the treatment groups, as usually occurs in observational studies. Conditional on the model, estimates of these coefficients may be unbiased, but in the usual situation in which the single model selected is wrong, then estimates will be biased. However, most studies in this field omit from their analysis the selection of variables that are explanatory of treatment outcomes. The use of a single model may ignore the question of model uncertainty and thus lead to underestimation of the uncertainty concerning quantities of interest. In fact, the full model, including all control variables, would have a poorer predictive capacity than the true model when some of the covariate effects are zero. In this case, the uncertainty about the prediction may also be overestimated.

In this paper, we examine different methods for variable selection from a Bayesian perspective. The Bayesian approach to model selection and to accounting for model uncertainty overcomes the difficulties encountered with the classical approach, based on *p*-values. Bayesian estimation expresses all uncertainty, including uncertainty about the correct model, in terms of probability. Therefore, we can directly estimate the subsequent probability of a model, or the probability that a covariate is included in the real model. Moreover, the estimation process for the Bayesian variable selection problem is, in principle, straightforward, with all results following directly from elementary probability theory, the definition of conditional probability, Bayes’ theorem and the law of total probability [13–15].

Raftery *et al*. [16] pioneered model selection and accounting for model uncertainty in linear regression models. The tutorial on Bayesian Model Averaging (BMA) given by Hoeting *et al*. [14] provides a historical perspective on the combination of models and gives further references. Although many papers have been published about Bayesian model selection in applied economic models [17–21, among others], there are few examples of this methodology in the health economics field. Recently, Negrín and Vázquez-Polo [22] showed that the BMA methodology can potentially be used to guide the practitioner in choosing between models, and proposed the use of the Fractional Bayes Factor (FBF) for model comparison. In the present paper, we extend this study, to compare four alternative Bayesian procedures for model selection: the Bayes Information Criterion (BIC), the Intrinsic Bayes Factor (IBF), the Fractional Bayes Factor (FBF) and a novel procedure based on intrinsic priors [23–26]. The model selection is also applied to subgroup analysis and within the net benefit regression framework.

BMA has also been studied to account for uncertainty in non-linear regression models. For example, Conigliani and Tancredi [27] proposed the use of Bayesian Model Averaging to model the distribution of costs as an average of a set of highly skewed distributions, while Jackson *et al*. [28] applied BMA in a long-term Markov model using Akaike’s Information Criterion (AIC) and BIC approximations. These authors concluded that the BIC method is more suitable when there is believed to be a relatively simple true model underlying the data. Jackson *et al*. [29] included uncertainty about the choice between plausible model structures for a Markov decision model, using Pseudo Marginal Likelihood (PML) and the Deviance Information Criterion (DIC) for model comparison.

Bayesian statistics are commonly employed in the field of cost-effectiveness analysis, with Spiegelhalter *et al*. [30] and Jones [31] being among the first to discuss the Bayesian approach for statistical inference in the comparison of health technologies. Since then, many studies have used the Bayesian approach to compare treatment options by means of cost-effectiveness analysis [32–38].

The rest of this paper is organized as follows. Section 2 briefly reviews the Bayesian variable selection procedures, and presents the four methods that are compared in this paper. Section 3 describes a simulation study carried out to validate the different methods. A practical application with real data is shown in Section 4, together with some possible applications of variable selection in the economic evaluation context. Finally, in Section 5, the work is summarised and some conclusions drawn.

## 2. Methodological Concepts

### 2.1. Bayesian Normal Linear Regression Model

In this paper we focus on the problem of Bayesian variable selection for linear regression models. Section 4 provides a brief explanation of the model considered for cost-effectiveness analysis, but we first present the methodological aspects involved in Bayesian variable selection, using the general linear regression model defined by the equation:

y=Xβ+u

or equivalently:

(1) $$y_{h}=\beta_{1}+\beta_{2}\cdot x_{2,h}+\beta_{3}\cdot x_{3,h}+\ldots +\beta_{k}\cdot x_{k,h}+u_{h},\;\;\;h=1,\ldots ,n,$$

where *y* = (*y*_1_,..., *y_h_*,..., *y_n_*)′ is a *n* vector of observations of the dependent variable. The design matrix *X* = (*x*_1_, *x*_2_,..., *x_n_*)′, with dimension (*n* × *k*) includes the exogenous variables in the sample where *x_h_* = (1, *x*_2,_*_h_*,..., *x_k_*_,_*_h_*), *h* =1,..., *n* for individual *h*. The vector *β* = (*β*_1_, *β*_2_,..., *β_k_*)′ ∈ ℝ*^k^* is the vector of unknown regression coefficients, and *u_h_* is the error term which is assumed to be independent and normally distributed with mean 0 and variance σ^2^.

Assuming the above hypothesis about *u*’s, the likelihood of β and σ^2^ is given by:

(2) $$\ell (y|\beta ,\sigma^{2})∼N(X\beta ,\sigma^{2}I_{n}),$$

where *I_n_* denotes the *n* × *n* identity matrix.

The usual choice of prior distribution parameter in the context of linear regression models is the conjugate normal-inverse-gamma prior [15]. The normal–inverse-gamma distribution is adopted as the prior distribution for the vector coefficient (β) and variance term (σ^2^):

(3) $$\pi (\beta ,\sigma^{2})\propto {\left(\sigma^{2}\right)}^{-(d+k+2)/2}\text{exp}[-[{\left(\beta -\beta^{0}\right)}^{\prime }V^{-1}(\beta -\beta^{0})+a]/(2\sigma^{2})],$$

with hyperparameters β^0^, *V*_1_, *a* and *d*.

Combining likelihood and prior distribution through Bayes’ theorem, we obtain the posterior distribution of β and σ. This posterior is also normal–inverse-gamma, as shown in [39]:

(4) $$\pi (\beta ,\sigma^{2}|y,X)\propto {\left(\sigma^{2}\right)}^{-(d+k+2+n)/2}\text{exp}[-[{\left(\beta -\beta^{*}\right)}^{\prime }{\left(V^{*}\right)}^{-1}(\beta -\beta^{*})+a^{*}]/(2\sigma^{2})],$$

where:

$$\begin{array}{c} V^{*}={\left(V^{-1}+X'X\right)}^{-1},\\ \beta^{*}{\left(V^{-1}+X'X\right)}^{-1}(V^{-1}\beta^{0}+X'y),\\ a^{*}=a+(\beta^{0})'V^{-1}\beta^{0}+y'y-(\beta^{*})'V^{-1}\beta^{*}. \end{array}$$

The marginal posterior distribution of β is obtained by integrating out σ using the integration from Equation (4). Therefore, we have:

$$\pi (\beta |y,X)\propto {\left[1+(\beta -\beta^{*})'{\left(a^{*}V^{*}\right)}^{-1}(\beta -\beta^{*})\right]}^{-\frac{d+k+n}{2}},$$

which is a Student *t*–distribution with *d*+*n* degrees of freedom and hyperparameters β^*^, a^*^ and V^*^, with mean and variance–covariance matrix given by β^*^ and $\frac{a^{*}}{d+n-2}$ · V^*^, respectively.

Using conjugacy properties, it can be obtained directly that the conditional distribution of σ^2^ given β is an inverse-gamma, *IG*(A,B), with parameters:

$$\begin{array}{c} A=(\beta -\beta^{*})'{\left(V^{*}\right)}^{-1}(\beta -\beta^{*})+a^{*},\\ B=d+k+n. \end{array}$$

### 2.2. Bayes Factors and Posterior Model Probabilities

Suppose that we are comparing *q* models for data *x*:

$$M_{i}:X∼f_{i}(x|\theta ),\;i=1,\ldots ,q,$$

where θ*_i_* is an unknown parameter. Assume, moreover, that we have prior distributions, *π_i_* (*θ_i_*), *i* =1,..., *q*, for the unknown parameters, and consider the marginal densities of *x* :

$$m_{i}(x)=\int f_{i}(x|\theta_{i})\pi_{i}(\theta_{i})d\theta_{i}.$$

The *Bayes factor* for comparing models *M_j_* and *M_i_* is given by:

(5) $$B_{\text{ji}}=\frac{m_{j}(x)}{m_{i}(x)}=\frac{\int f_{i}(x|\theta_{j})\pi_{j}(\theta_{j})d\theta_{j}}{\int f_{i}(x|\theta_{i})\pi_{i}(\theta_{i})d\theta_{i}}.$$

The Bayes factor is often interpreted as the “odds provided by the data for *M_j_* over *M_i_*”. Thus *B_ji_*=5 would suggest that the data favour *M_j_* over *M_i_* at odds of 5 to one. Alternatively, *B_ji_* is sometimes called the “weighted likelihood ratio of *M_j_* to *M_i_*”, with the priors being the “weighting functions”. From Equations (2) and (3), it follows that the Bayes factor in favour of model *j* versus model *i* for linear models has the expression:

(6) $$B_{\text{ji}}=\frac{{\left|V_{i}\right|}^{1/2}{\left|V_{j}^{*}\right|}^{1/2}}{{\left|V_{j}\right|}^{1/2}{\left|V_{i}^{*}\right|}^{1/2}}\cdot {\left(\frac{a_{i}^{*}}{a_{j}^{*}}\right)}^{(d+n)/2}.$$

If prior probabilities of the models, *π*(*M_i_*), *i* = 1,..., *q*, are available, then one can compute the posterior probabilities of the models as:

(7) $$\pi (M_{i}|x)=\frac{\pi (M_{i})m_{i}(x)}{\sum_{j=1}^{q}\pi (M_{j})m_{j}(x)}={\left(\sum_{j=1}^{q}\frac{\pi (M_{j})}{\pi (M_{i})}\cdot B_{ji}\right)}^{-1}.$$

For a uniform prior on the models, $\pi (M_{i})=\frac{1}{q}$, *i =* 1*,..., q,* expression (7) becomes:

(8) $$\pi (M_{i}|x)=\frac{1}{\sum_{j=1}^{q}B_{ji}}=\frac{m_{i}(x)}{\sum_{j=1}^{q}m_{j}(x)}.$$

where *π*(*M_i_* | *x*) represents the posterior probability of model *i*. Using this posterior probability, we can observe the models with the highest probabilities or compute the probability of inclusion of a covariate as the sum of the posterior probabilities of all the models that include this covariate.

### 2.3. Objective Bayesian Methods and Model Selection

Observe that the variable selection problem is by its nature a model selection problem, in which we must choose one model from among 2*^k^* possible submodels of the above full one (1). It is common to set *β*_1_≠ 0 to include the intercept in any model. In this case the number of possible submodels is 2*^k^*^−1^.

A model containing no regressors but only the intercept is denoted as *M*^1^, and a model containing *i* regressors, *i* ≤ *k*, is denoted as *M^i^*. There are $\left(\begin{array}{l} k\\ i \end{array}\right)$ models *M^i^* and for each one there is a specific data set and a design matrix. Note that any model is nested within the full model and that the intercept-only model *M*^1^ is nested within any model *M^i^*.

As shown in the previous section, Bayesian analysis permits the inclusion of prior information about the parameters. However, the use of prior information becomes problematic for variable selection. A model with *k* covariates requires the elicitation of 2*^k^* submodels that include from zero to k covariates, that is, *k*·2*^k^*^−1^ coefficients must be elicited. Partial solutions such as eliciting only the coefficients for the full model, using these prior distributions for the remaining models, are not appropriate because the meaning of common parameters can change from one model to another.

A possible solution is to carry out a Bayesian analysis assuming noninformative prior distributions. However, it is well known that the use of improper noninformative priors is not possible in model selection. Indeed, let us assume that a conventional improper prior is used for a generic model *M_i_*,

$$\pi (\beta ,\sigma^{2}\propto \sigma^{-2}.$$

This improper prior is equivalent to the prior distribution in Expression (3), setting *V^−1^* = 0, *a* = 0 and *d* = −*k*. The Bayes factor, *B_ji_* given by equation (6) is not well-defined for improper priors because of the terms |*V_i_*|^1/2^ and |*V_j_*|^1/2^, both of which are infinite.

Alternative procedures for variable selection have recently been developed. In this paper, we compare four of these procedures: Bayesian Information Criterion (BIC), Intrinsic Bayes factor (IBF), Fractional Bayes factor (FBF) and the most recent technique, one that provides an objective Bayesian solution based on intrinsic priors [23–26,40–41]. An objective Bayesian solution seems to be particularly suitable for this problem since little subjective prior information can be expected on the regression coefficient of a regressor when we do not know whether it should be included in the model.

#### 2.3.1. Bayesian Information Criterion (BIC)

The BIC approximation, also known as Schwarz’s information criterion, is a widely used tool in model selection, largely due to its computational simplicity and effective performance in many modelling frameworks. The derivation of BIC [42] establishes the criterion as an asymptotic approximation to a transformation of the Bayesian posterior probability of a candidate model. It has the advantage of simplicity and avoids the need to specify an explicit prior for each model [43–47]. The usual BIC for linear models has the simple expression:

(9) $$B_{ji}={\left(\frac{{e^{\prime }}_{i}e_{i}}{{e^{\prime }}_{j}e_{j}}\right)}^{n/2}.n^{(k_{i}-k_{j})/2},$$

where *e_i_*′*e_i_* and *e_j_*′*e_j_* are the residual sums of squares under models *i* and *j*, respectively, and *k_i_*, *k_j_* is the dimension of the models.

#### 2.3.2. Intrinsic Bayes Factor (IBF)

The general strategy for defining the IBF starts with the definition of a proper and minimal training sample, which is to be viewed as a subset of the entire data *x*. Because we will consider a variety of training samples, these are indexed by *l*. The standard use of a training sample to define the Bayes factor is to use *x*(*l*) to convert the improper π*_i_*(θ*_i_*) into a proper posterior π*_i_*(θ*_i_*|*x*(*l*)), and then use the latter to define Bayes factors for the remaining data. The result, for comparing *M_j_* to *M_i_*, can be seen to be:

$$B_{ji}(l)=B_{ji}^{N}(x).B_{ij}^{N}(x(l)),$$

where:

(10) $$B_{ji}^{N}=B_{ji}^{N}(x)=\frac{m_{j}^{N}(x)}{m_{i}^{N}(x)}\;\text{and}\;B_{ij}^{N}(l)=B_{ij}^{N}(x(l))=\frac{m_{i}^{N}(x(l))}{m_{j}^{N}(x(l))},$$

are the Bayes factors that would be obtained for the full data *x* and training sample *x*(*l*), respectively, if one were to blindly use *π_i_^N^* and *π_j_^N^*.

While *B_ji_*(*l*) no longer depends on the arbitrary scales of *π_j_^N^* and *π_i_^N^*, it does depend on the arbitrary choice of the (minimal) training sample *x*(*l*). To eliminate this dependence and to increase stability, we average the *B_ji_*(*l*) over all possible training samples *x*(*l*), *l* = 1,…,*L*. A variety of different averages are possible; here we consider only the arithmetic mean IBF, defined as:

(11) $$B_{ji}^{\text{mean}}=B_{ji}^{N}\cdot \frac{1}{L}\sum_{l=1}^{L}B_{ij}^{N}(l).$$

Different noninformative priors can be considered. Here we consider the improper reference priors of the form:

$$\pi_{j}^{N}(\beta_{j},\sigma_{j}^{2})=\sigma_{j}^{-2}.$$

For these priors, a minimal training sample (*y*(*l*), *x*(*l*)) is a sample of size *m* such that all (*X_j_*′*X*_j_) are nonsingular. Then, the Bayes factor is:

(12) $$B_{ji}^{N}=\pi^{(k_{j}-k_{i})/2}.\frac{\Gamma ((n-k_{j})/2)}{\Gamma ((n-k_{i})/2)}.\frac{{\left|{X^{\prime }}_{i}X_{i}\right|}^{1/2}}{{\left|{X^{\prime }}_{j}X_{j}\right|}^{1/2}}.\frac{{e^{\prime }}_{i}e_{i}^{(n-k_{i})/2}}{{e^{\prime }}_{j}e_{j}^{(n-k_{j})/2}}.$$

The formula of *B_ij_^N^* (*l*) is given by the inverse of this expression replacing *n*, *X_i_*, *X_j_*, *e_i_*′*e_i_* and *e_j_*′*e_j_* by *m*, *X_i_* (*l*), *X_j_* (*l*), *e_i_*′*e_i_* (*l*) and *e_j_*′*e_j_* (*l*), respectively. By using the above expressions to calculate *B_ji_*^mean^ we obtain the expression:

(13) $$B_{ji}^{\text{mean}}=A\cdot \frac{{\left|{X^{\prime }}_{i}X_{i}\right|}^{1/2}}{{\left|{X^{\prime }}_{j}X_{j}\right|}^{1/2}}\cdot \frac{{\left({e^{\prime }}_{i}e_{i}\right)}^{(n-k_{i})/2}}{{\left({e^{\prime }}_{j}e_{j}\right)}^{(n-k_{j})/2}}\cdot \frac{1}{L}\sum_{l=1}^{L}\frac{{\left|{X^{\prime }}_{j}(l)X_{j}(l)\right|}^{1/2}}{{\left|{X^{\prime }}_{i}(l)X_{i}(l)\right|}^{1/2}}\cdot \frac{{\left({e^{\prime }}_{j}e_{j}(l)\right)}^{(n-k_{j})/2}}{{\left({e^{\prime }}_{i}e_{i}(l)\right)}^{(n-k_{i})/2}},$$

where:

$$A=\frac{\Gamma ((n-k_{j})/2)}{\Gamma ((n-k_{i})/2)}.\frac{\Gamma ((m-k_{i})/2)}{\Gamma ((m-k_{j})/2)}.$$

For a detailed derivation of these Bayes factors for the linear model see [40].

#### 2.3.3. Fractional Bayes Factor (FBF)

The fractional Bayes factor [48] is based on a similar understanding to that underlying the IBF. It uses a proportion or fraction, b (training sample), of data to obtain an initial informative posterior distribution of the parameter for each model. The remaining 1–*b* fraction of the likelihood is used for model discrimination. The minimal fraction is used to obtain the fractional Bayes factor, defined as the ratio between the minimal training sample described in 2.3.2 and *n*. The expression of the fractional Bayes factor for the linear regression model is given in [39]:

(14) $${\text{FBF}}_{\text{ji}}=\frac{\Gamma ((nb-k_{i})/2)}{\Gamma ((nb-k_{j})/2)}\cdot \frac{\Gamma ((n-k_{i})/2)}{\Gamma ((n-k_{j})/2)}\cdot {\left(\frac{{e^{\prime }}_{i}e_{i}}{{e^{\prime }}_{j}e_{j}}\right)}^{n(1-b)/2}.$$

#### 2.3.4. Bayes Factor for Intrinsic Priors

This method is based on the use of intrinsic priors, an approach that was introduced by Berger and Pericchi [40] to overcome the difficulty arising with conventional priors in model selection problems. It has been studied by Moreno *et al*. [41], among others. Justifications for the use of intrinsic priors for model selection have been given by Berger and Pericchi [49]. Design considerations about this method are made in [50], and an application is shown in [24].

The method is described as follows. Using the definition of the matrix *X* defined in Section 2.1, we consider all sub-matrices *X_j_*⊂*X* containing *j*–regressors, for *j* = 1,...,*k*. The Bayes factor for intrinsic priors [50] is then computed as follows:

(15) $$B_{j1}=\frac{2{\left(j+1\right)}^{(j-1)/2}}{\pi }\cdot \int_{0}^{\pi /2}\frac{{\left(\text{sin}\phi \right)}^{j-1}{\left[n+(j+1){\text{sin}}^{2}\phi \right]}^{(n-j)/2}}{{\left(nB_{j}+(j+1){\text{sin}}^{2}\phi \right)}^{(n-1)/2}}d\phi ,$$

where ${\text{B}}_{j}=\frac{y^{\prime }(I-H_{j})y}{ns_{y}^{2}}$, *H_j_* = *X_j_* *(X_j_*′*X_j_)*^−1^ *X_j_*′, and *s_y_*^2^ is the sample variance of variable *y*.

The posterior probability of model *M_j_* is given by the expression:

(16) $$\text{Pr}(M_{j}|x)=\frac{B_{j1}}{1+\sum_{i\neq 1}B_{i1}}.$$

## 3. A Simulated Experiment

In this section we validate the variable selection methods proposed in the previous section for linear regression models, using simulated data. Our aim is not to study the differences between methods in a wide variety of circumstances, but rather to show how the models perform and how large the posterior probability of inclusion must be to suggest that a variable, such as treatment, influences the outcome.

We simulate six variables (*x*_1_, ..., *x*_6_) following the distributions described in Table 1. The first three variables are simulated from normal distributions, the next two are discrete variables following a Bernoulli process, and the last one is distributed as a Poisson distribution. The parameters of the simulated distributions are shown in Table 1. The dependent variable *y* is obtained as a linear combination of three of them (*x*_2_, *x*_4_ and *x*_6_). The expression used to obtain *y* is also shown in Table 1.

We evaluate the results of the different methods for three different sample sizes *n*_1_ = 30, *n*_2_ = 100, *n*_3_ = 300. For every method, we estimate the probability of all possible models. To calculate the probability of inclusion for each covariate, we sum the probabilities of the different models that include this covariate. The results are shown in Tables 2, 3 and 4, respectively.

Some conclusions can be drawn from these results. For our standard sampling model, the four models tested obtain very similar and accurate results. The BIC and the Bayes Factor for intrinsic priors seem to be slightly better than the others, providing higher probabilities of inclusion for the explanatory variables (*x*_2_, *x*_4_ and *x*_6_). For the smallest sample size (*n*_1_ = 30), the methods only estimate around 55% of inclusion for the binary variable *x*_4_. As expected, with large sample sizes the probabilities of inclusion for the relevant covariates are close to one and the results for the four methods are very similar.

The simulation results suggest that the probability of a true covariate being accepted depends on the distribution of the covariate and the sample size. With a small sample size, covariates with a probability of inclusion greater than 50% could be judged to truly affect the outcome. However, with sample sizes exceeding 300, the required probability of inclusion should be more than 80%.

As well as the probability of inclusion, using the Bayesian variable selection described in Section 2 it is possible to estimate the posterior probability of each model. The selection of the model with the highest probability can lead to erroneous conclusions being drawn, because this ignores the probability associated with the other models. Of course, this method would be appropriate when the posterior probability of the best model is very high. In our simulated experiment, the true model was always found to be the model with the highest posterior probability for the selection model based on BIC, although this probability varied for different sample sizes (22.32%, 33.08% and 77.61%, for the sample sizes 30, 100 and 300, respectively). The IBF obtains similar results to those of the BIC model. However, the FBF and the procedure based on intrinsic priors produce the model that includes the covariates *x*_2_, *x*_3_, *x*_4_ and *x*_6_ as the most probable model for a sample size of 100, although the posterior probability is very similar to that obtained by the real model (32.83% *vs*. 32.35% for the FBF, and 27.96% *vs*. 25.34% for the procedure based on intrinsic priors).

## 4. Practical Application

We analyzed the usefulness of these methods for variable selection with a real clinical trial, comparing two highly-active antiretroviral treatment protocols applied to asymptomatic HIV patients [51]. Each treatment combines three drugs and we denote them as control treatment (d4T + 3TC + IND) and new treatment (d4T + ddl + IND).

We obtained data on effectiveness, QALYs using EuroQol-5D [52], and on the direct costs for the 361 patients included in the study. The QALYs were calculated as the area above/below the utility value. This approach to QALYs takes into account the differences in baseline utility values [53]. All patients kept a monthly diary for six months to record resource consumption and quality of life progress.

As control variables we considered the *age*, the *gender* (value 0 for a male patient and value 1 for a female) and the existence of any concomitant illness (*cc1* with a value of 1 if a concomitant illness is present, and 0 otherwise, and *cc2* with a value of 1 if two or more concomitant illnesses are present, and 0 otherwise). The concomitant illnesses considered were hypertension, cardiovascular disease, allergies, asthma, diabetes, gastrointestinal disorders, urinary dysfunction, previous kidney pathology, high levels of cholesterol and/or triglycerides, chronic skin complaints and depression/anxiety. The time (in months) elapsed from the start of the illness until the moment of the clinical trial was also included in the model. Finally, the treatment was included as a dichotomous variable (*T*) that was assigned a value of 1 if the patient received the (d4T + ddl + IND) treatment protocol and a value of 0 if the (d4T + 3TC + IND) treatment was applied. Table 5 summarizes the statistical data.

Our aim is to explain the effectiveness and cost as a function of the treatment received, and controlled by covariates. The full model includes all the control variables and is given by:

(17) $$E=\beta_{0}+\beta_{1}\cdot \text{age}+\beta_{2}\cdot \text{gender}+\beta_{3}\cdot \text{ccl}+\beta_{4}\cdot \text{cc2}+\beta_{5}\cdot \text{start}+\beta_{T}\cdot T+u,$$

(18) $$C=\delta_{0}+\delta_{1}\cdot \text{age}+\delta_{2}\cdot \text{gender}+\delta_{3}\cdot \text{ccl}+\delta_{4}\cdot \text{cc2}+\delta_{5}\cdot \text{start}+\delta_{T}\cdot T+v,$$

The joint likelihood for β,δ,Σ is defined by a multivariate normal distribution:

(19) ℓ(E,C|β,δ,Σ)∼N((Xβ,Xδ),Σ),

where $\Sigma =\left(\begin{array}{cc} \sigma_{u}^{2} & \sigma_{uv}\\ \sigma_{uv} & \sigma_{v}^{2} \end{array}\right)$.

Effectiveness and cost are not independent and so we allow some correlation between the error terms of both equations. However, model selection is computationally complex when bivariate distributions are considered [54,55]. Posterior probabilities cannot be calculated analytically, and Markov Chain Monte Carlo (MCMC) techniques are required. For this reason, we performed Bayesian variable selection for each equation separately (assuming σ*_uv_* = 0). Although the proposed model allows for the existence of correlation between effectiveness and costs, in this practical application, as in many others, this correlation is low (the sample correlation is −0.0006). The final model is estimated assuming this correlation after calculating the probabilities of inclusion for each covariate [8,10].

**Figure 1.**
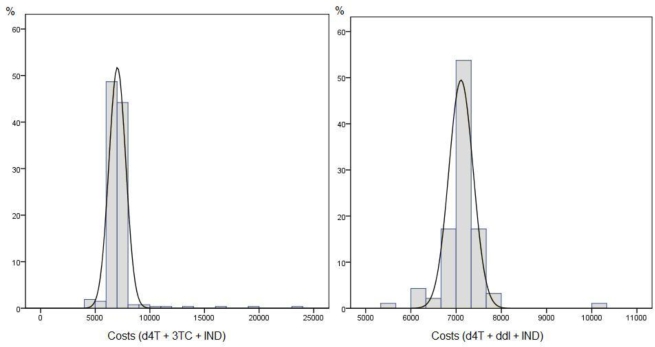
Histogram of costs.

Cost transformations, as a logarithm, are often proposed to take account of the right skewing which is often present. However, this transformation poses a difficulty for the interpretation of the results, because due to the robustness of linear methods, costs are not transformed in the presence of low levels of right-skewing, as has been shown by Willan *et al*. [8] with simulated data. Log-normal, gamma or other skewed distributions would be more suitable for very skewed data [11]. Selection and averaging between models with non-normal distributions are discussed in [27] although covariates are not considered in the latter paper. Figure 1 shows the costs histogram for each treatment in our practical application.

The results of variable selection under the four methods and for both equations, effectiveness and cost, are shown in Table 6.

Only one control variable was found to have relevant explanatory power (*cc*2 in the effectiveness equation). The posterior probabilities for the other control variables were always below 30%. In this example, the analysis based on the full model would achieve very different conclusions from those obtained by the real model.

The most probable model for effectiveness includes only one control variable in the equation (*cc*2). The probability associated with this variable varies from 56.02%, for the BIC criterion, to 48.70% for the procedure based on intrinsic priors.

The most probable model for cost does not include any control variables. The posterior probabilities of this model are 67.47% for BIC, 15.36% for IBF, 50.69% for FBF and 61.94% for the procedure based on intrinsic priors. Results for the most probable model are shown in Table 7.

The aim of a regression framework applied to cost-effectiveness analysis is to calculate the incremental effectiveness and incremental cost by estimating the coefficient of the treatment indicator in the effectiveness and cost equations, respectively. For this reason, we also include the treatment indicator in the final model. The incremental effectiveness is estimated as being 0.001714, with a posterior 95% Bayesian interval (−0.01341, 0.01699). The incremental cost is estimated as being 164.1 euros, with a posterior 95% Bayesian interval (−215.9, 543.6).

### 4.1. Probability of the Inclusion of the Treatment in the Regression Model

One probability that deserves special mention is the posterior probability of the treatment. The aim of cost-effectiveness analysis is to estimate the incremental effectiveness and incremental cost of a new treatment versus the control. The inclusion of the treatment indicator in the equations of cost and effectiveness allows the analyst to estimate the incremental effectiveness and cost from their respective coefficients (β*_T_* and δ*_T_*). In this practical application, we show that the probabilities of inclusion of the treatment indicator in the effectiveness and cost equations are very low (0.05304 and 0.07019, respectively for the BIC method). The conclusion of this result is that the treatment indicator is not a good predictor of the effectiveness or cost, as the incremental effectiveness and cost are close to zero. However, in the model shown in Table 7, the treatment indicator is included in the final model, ignoring model uncertainty. From the width of the Bayesian intervals of the treatment indicator in both equations, we conclude that differences in incremental effectiveness and cost are not relevant between different treatments, and that a point estimation based on the posterior mean would be biased.

We can estimate only the most probable model, but in our example this model only has a probability close to 50% and the estimation based on this model ignores the uncertainty about the other models. BMA [14,56,57] provides a natural Bayesian solution to estimation in the presence of model uncertainty. The estimation of the coefficients is obtained as a combination of the coefficients estimated for each model, weighted by the posterior probability of each model. Therefore, the mean of the incremental effectiveness (the expression is analogous for the incremental cost) is obtained by the expressions:

(20) $$E(\beta_{T}|X)=\sum_{j=1}^{q}E(\beta_{T}|M_{j},X)\cdot \text{Pr}(M_{j}|X).$$

An expression for the posterior variance of β*_T_* is given by Leamer [58]:

(21) $$V(\beta_{T}|X)=\sum_{j=1}^{q}V(\beta_{T}|M_{j},X)\cdot \text{Pr}(M_{j}|X)+\sum_{j=1}^{q}{\left(E(\beta_{T}|M_{j},X)-E(\beta_{T}|X)\right)}^{2}\cdot \text{Pr}(M_{j}|X).$$

Negrín and Vázquez-Polo [22] described an application of BMA in a cost-effectiveness analysis using the same data set. The estimated incremental effectiveness was 0.00141, with a standard deviation of 0.0047, for the full model and 0.00018, with a standard deviation of 0.00162, when BMA methodology was applied. The incremental cost was 164.3125 euros, with a standard deviation of 196.5101, for the full model and 98.9067, with a standard deviation of 170.4027, for the BMA model. It is important to point out that these results are not fully comparable with those given in this paper because [22] included prior information on the models.

### 4.2. Subgroup Analysis

Subgroup analysis is becoming a relevant aspect of economic evaluation [8,11]. For example, suppose that we are interested in determining whether a certain subgroup has the same incremental effectiveness or incremental cost as a reference subgroup. The regression model allows for subgroup analysis by including the interaction between the subgroup indicator and the treatment indicator. The existence of subgroups is studied by analyzing the statistical relevance of this interaction. Classical hypothesis tests have been proposed for this item, but Bayesian variable selection allows a natural quantity to be estimated, as this is the posterior probability of inclusion.

As an example, suppose that we are interested in studying whether there are differences in treatment results between males and females. To analyze the relevance of the subgroup, we include the interaction *gender* × *T* as an explanatory covariate of effectiveness and cost.

The posterior probability of inclusion of the interaction in the effectiveness equation varies from the 9.1844% of the BIC method to the 17.2234% of the intrinsic priors method. In view of these results, we cannot accept the existence of a subgroup in the effectiveness model. Analogously, the posterior probability of inclusion in the cost equation varies from the 19.0928% of the BIC method to the 49.4215% of the IBF method. In this case, the probability of there being a subgroup in the cost model is higher, although it is always below 50%.

It is important to recall that in conventional frequentist clinical trial protocols, it is mandatory to specify any intended subgroup analysis in advance, and drug regulatory agencies are very wary of allowing claims for subgroup effects, because of the risk of data dredging [59–61]. In Bayesian analysis, the corresponding guidance should be that the prior distributions for the coefficients of these interaction terms must be specified to reflect genuine belief about how large such subgroup effects might realistically be, based on the existence and plausibility of appropriate biological mechanisms [62]. We have shown in this subsection that Bayesian variable selection methodology can be used for exploratory subgroup analysis.

### 4.3. Net Benefit Regression Framework

The Net Benefit regression framework was introduced to facilitate the use of regression tools in economic evaluation [7]. Net benefit regression uses as the dependent variable the net benefit, *z* = *R*·*e*–*c*, where *e* refers to the effectiveness, *c* refers to the cost and *R* is the ceiling ratio, which can be interpreted as the decision maker’s willingness to pay for an increment in effectiveness. The equation should include an indicator of the treatment provided. The coefficient of this indicator is equal to the difference in mean net benefit for the new and control treatments. It has been shown [7,8] that when this difference is greater than zero then the incremental net benefit is positive and the new treatment is preferred.

A difficulty with the net benefit regression framework is that the net benefit depends upon the decision maker’s willingness to pay (*R*), a value that is not known from the cost and effect data. Thus, it is necessary to estimate a new equation for each value of *R* considered. The variable selection procedures can be applied to this framework. As an example, Table 8 shows the results of the variable selection with the intrinsic priors method for three different values of *R* (*R*_1_ = 0, *R*_2_ = 50,000 and *R*_3_ = 100,000). As expected, the probabilities of inclusion for *R* = 0 coincide with the cost equation in Table 6. For greater values of *R*, the probabilities of inclusion will be more similar to those obtained for the effectiveness equation.

## 5. Conclusions

Linear regression is often used to account for the cost and effectiveness of medical treatment. The covariates may include sociodemographic variables, such as age, gender or race; clinical variables, such as initial health status, years of treatment or the existence of concomitant illnesses; and a binary variable indicating the treatment received. The coefficient of the treatment variable for the effectiveness and cost regression can be interpreted as the incremental effectiveness and incremental cost, respectively. Several recent studies have been made of the usefulness of including covariates in cost-effectiveness analysis, using approaches based on incremental cost-effectiveness or incremental net benefit [7–8,11]. These studies were carried out in a frequentist framework, while Vázquez-Polo *et al.* [10] developed a similar analysis from a Bayesian perspective.

However, most studies assume only one model, usually the full one. In so doing, they ignore the uncertainty in model selection. In the present paper, we consider the four most important alternative Bayesian variable selection methods for estimating the posterior probability of inclusion of each covariate. A simulation exercise shows the performance of these methods with linear regression models, and we conclude that all of them have high and similar levels of accuracy. It has long been known that when sample sizes are large, the BIC criterion provides a reasonable preferred model, in view of its straightforward approximation procedure and the use of an implicit prior. As the four proposed methods in the paper do not give widely varying conclusions in the real example, the choice of which of these criteria to use depends on the purpose of the model assessment [28]. In our practical application, we considered a moderately large sample, and thus the BIC measure yielded an easily computable quantity with no need for computer-intensive calculations. For small sample sizes, we recommend the use of IBF or BF under intrinsic priors, due to the good properties presented by these methods: the measures are completely automatic Bayes factors, IBFs are applicable to nested as well as nonnested models, and they are invariant to univariate transformations of the data, among other advantages [40]. The Bayesian procedures for variable selection with intrinsic priors are consistent and, furthermore, Lindley’s paradox (*i.e*., a point null hypothesis on the normal mean parameter is always accepted when the variance of the conjugate distribution tends to infinity) does not arise. We believe, in accordance with Casella *et al*. [25] that ‘intrinsic priors provide a type of objective Bayesian prior for the testing problem. They seem to be among the most diffuse priors that are possible to use in testing, without encountering problems with indeterminate Bayes factors, which was the original impetus for the development of Berger and Pericchi [40]. Moreover, they do not suffer from “Lindley’s paradox” behavior. Thus, we believe they are a very reasonable choice for experimenters looking for an objective Bayesian analysis with a frequentist guarantee.’

All the Bayesian model selection procedures presented enable the estimation of the posterior probability of each possible model and the probability of inclusion of each covariate. When the posterior probability of the “best” model is reasonably high, the use of this model is accurate. However, when the number of models compared is large, then the associated probability of the “best” model might be low. In this case, the BMA strategy provides a more appropriate alternative.

Moreover, complementary analyses are possible with variable selection. Thus, the incremental effectiveness and incremental cost may be estimated using BMA. Here, we advocate BMA analysis as the most coherent way to estimate the quantities of interest under model uncertainty.

Another interesting application of variable selection is subgroup analysis. The regression model allows for subgroup analysis by the inclusion of the interaction between the subgroup indicator and the treatment indicator. The existence of subgroups is studied by analyzing the statistical relevance of this interaction; this is precisely the aim of variable selection.

One difficulty with the variable selection approach is the computational burden involved when the number of possible regressors *k* is large or when interactions are considered. Then, the number of models becomes so large that it is impossible to compute all the posterior probabilities. In this case, we need to resort to a stochastic algorithm to compute only the high posterior probability model. An example of such an algorithm is given by Casella and Moreno [23]. This difficulty is not inherent to any specific variable selection procedure but is shared by all existing procedures.

## Figures and Tables

**Table 1.** Simulation exercise. Distribution of the variables simulated.

Variable	Distribution	Variable	Distribution
*x*_1_	*N*(μ = 10,σ = 3)	*x*_4_	*Ber*(*p* = 0.7)
*x*_2_	*N*(μ = 5,σ = 1)	*x*^5^	*Ber*(*p* = 0.2)
*x*_3_	*N*(μ = 0,σ = 3)	*x*_6_	*P*(λ = 4)

*y* = 5 + 3·*x*_2_ +7 · *x*_4_–4· *x*_6_ + *N*(0,8)			

**Table 2.** Bayesian variable selection. Simulation exercise (*n* = 30).

Variable	BIC	IBF	FBF	Intrinsic priors

Intercept	1	1	1	1
*x*_1_	0.37917	0.31609	0.38295	0.43725
*x*_2_	0.96490	0.92767	0.92733	0.94164
*x*_3_	0.36978	0.34273	0.36931	0.42703
*x*_4_	0.55726	0.51189	0.51474	0.55564
*x*_5_	0.16435	0.17051	0.19437	0.25248
*x*_6_	0.99619	0.98874	0.98653	0.99076

**Table 3.** Bayesian variable selection. Simulation exercise (*n* = 100).

Variable	BIC	IBF	FBF	Intrinsic priors

Intercept	1	1	1	1
*x*_1_	0.30080	0.29285	0.27802	0.41913
*x*_2_	0.99876	0.99802	0.99806	0.99848
*x*_3_	0.48868	0.47003	0.46857	0.59544
*x*_4_	0.99975	0.99958	0.99956	0.99965
*x*_5_	0.09133	0.08811	0.08191	0.16460
*x*_6_	1.00000	1.00000	1.00000	1.00000

**Table 4.** Bayesian variable selection. Simulation exercise (*n* = 300).

Variable	BIC	IBF	FBF	Intrinsic priors

Intercept	1	1	1	1
*x*_1_	0.07372	0.07362	0.08469	0.12227
*x*_2_	1.00000	1.00000	1.00000	1.00000
*x*_3_	0.07476	0.07545	0.08584	0.12404
*x*_4_	1.00000	1.00000	1.00000	1.00000
*x*_5_	0.09452	0.09349	0.10848	0.15315
*x*_6_	1.00000	1.00000	1.00000	1.00000

**Table 5.** Statistical summary of costs, effectiveness and patient characteristics: mean and standard deviation (in parenthesis).

	d4T + 3TC + IND	d4T + ddl + IND
Effectiveness (QALYs)	0.0113899 (0.0378566)	0.0123387 (0.0347704)
Cost (euros)	7142.44 (1573.98)	7307.26 (1720.96)
Age (years)	35.26 (7.36)	33.95 (6.77)
Gender (1-female, 0-male)	29%	27%
cc1	27%	32 %
cc2	11%	8%
Start	79.38 (92.32)	77.54 (102.19)
*n*	268	93

**Table 6.** Bayesian variable selection. Real data.

Effectiveness	BIC	IBF	FBF	Intrinsic priors

Intercept	1	1	1	1
*Age*	0.05617	0.08066	0.09352	0.10689
*gender*	0.07783	0.10726	0.12459	0.13894
*cc*1	0.16203	0.21744	0.25727	0.27340
*cc*2	0.86882	0.94134	0.92576	0.90300
*start*	0.05350	0.07795	0.08936	0.10253
*T*	0.05304	0.07403	0.08883	0.10203

Cost	BIC	IBF	FBF	Intrinsic priors

Intercept	1	1	1	1
*age*	0.05114	0.25580	0.09651	0.06950
*gender*	0.07101	0.32577	0.13125	0.09430
*cc*1	0.06615	0.29938	0.12237	0.08790
*cc*2	0.06134	0.28330	0.11346	0.08145
*start*	0.06056	0.29493	0.11313	0.08137
*T*	0.07019	0.31239	0.12952	0.09302

**Table 7.** Bayesian variable selection. Real data.

Effectiveness	Mean	s.d.	95% Bayesian Interval

Intercept	0.009151	0.004146	(0.000983, 0.0172)
*cc*2	0.01989	0.01121	(−0.001929, 0.04212)
*T*	0.001714	0.007757	(−0.01341, 0.01699)

Cost	Mean	s.d.	95% Bayesian Interval

Intercept	7142	98.52	(69481, 7334)
*T*	164.1	194.1	(−215.9, 543.6)

**Table 8.** Bayesian variable selection with intrinsic priors. Regression Net Benefit Framework.

Net Benefit	*R* = 0	*R* = 50,000	*R* = 100,000

Intercept	1	1	1
*age*	0.06950	0.09223	0.10047
*gender*	0.09430	0.17929	0.17130
*cc*1	0.08790	0.11447	0.17664
*cc*2	0.08145	0.75767	0.89573
*start*	0.08137	0.11231	0.11138
*T*	0.09302	0.09378	0.09521
